# Synchronicity of influenza activity within Phoenix, AZ during the 2015-2016 seasonal epidemic

**DOI:** 10.1186/s12879-017-2197-z

**Published:** 2017-01-31

**Authors:** James Tamerius, Jhobe Steadman, John Tamerius

**Affiliations:** 1 Department of Geographical and Sustainability Sciences, University of Iowa, Iowa City, IA USA; 2 Quidel Inc., San Diego, CA USA

**Keywords:** Influenza epidemic, Age-specific, Variability, Spatiotemporal variability, Scale

## Abstract

**Background:**

Variability in the timing of influenza epidemics has been observed across global and regional scales, but this variability has not been studied extensively at finer spatial scales. As such, the aim of this study was to test whether influenza cases were synchronized across sites and/or age-groups within a major city.

**Methods:**

We used influenza cases identified by rapid influenza tests from a network of clinics across Phoenix, AZ during the 2015–2016 influenza A season. We used a combination of KS tests and a bootstrapping approach to evaluate whether the temporal distribution of cases varied by site and/or age group.

**Results:**

Our analysis indicates that the timing of influenza cases during the 2015–2016 seasonal influenza epidemic were generally synchronized across sites and age groups. That said, we did observe some statistically significant differences in the timing of cases across some sites, and by site and age group. We found no evidence that influenza activity consistently begins or peaks earlier in children than in adults.

**Conclusions:**

To our knowledge, this is the first study to investigate differences in the intra-urban timing of influenza using influenza-specific case data. We were able to show evidence that influenza cases are not entirely synchronized across an urban area, but the differences we observed were relatively minor. It is important to understand the geographic scale at which influenza is synchronized in order to gain a better understanding of local transmission dynamics, and to determine the appropriate geographic scale that influenza surveillance data should be aggregated for prediction and warning systems.

## Background

Periodic epidemics of influenza characterize all human populations, yet there are significant differences in the timing of epidemics at global and regional scales. For instance, annual seasonal influenza epidemics in temperate regions of the southern and northern hemisphere are approximately 6 months out of phase. Further, although the timing of an epidemic within temperate regions of the same hemisphere occur at roughly the same time of the year, there can be substantial differences in their timing across countries and regions [1–7]. These differences have been related to workflow patterns [2], air travel [8] and environmental differences [7, 9]. Despite significant research on the spatial spread of influenza, existing studies have focused on global and regional spatial scales that span 100’s to 1000’s of km, and far less attention has been paid to variability of influenza within a single urban area. The paucity of studies focused on local scales is primarily due to a lack of data available that is sufficiently spatially-resolved and voluminous.

Another area of interest in influenza epidemiology is the synchronicity of cases across age-groups within the same population. Although there is evidence that local influenza activity tends to peak sooner in school aged children than in adults [10], the results of other studies have been mixed [11, 12]. For example, Schanzer et al. (2011) found that 10–19 and 20–29 year age groups peaked earlier for seasonal influenza than other age groups, but only the 10–19 age group peaked earlier during the 2009 pandemic. Further, Timpka et al. (2012) observed that cases in children occurred earlier than adults for A/H1N1 outbreaks, but no discernible differences across age groups were observed for seasonal A/H3N2 outbreaks. Differences in the timing of cases across age groups may be explained by differences in rates of physical and close contact between age groups [13], and may also be explained by differences in the underlying herd immunity across age groups that can be engendered by previous influenza exposures [14].

Here we use daily influenza A cases detected by rapid tests across a network of clinics in Phoenix, AZ during the 2015–2016 season to evaluate the synchronicity of cases across sites and age groups. We assume that the different clinics provide a measure of the local intensity of the epidemic by time, and significant deviations in the number of cases at a site from the expected number of cases indicates locally anomalous influenza activity. Understanding the strength of synchronicity in flu cases on small geographic scales may prove to be important to understand local transmission dynamics, and lead to more accurate and precise predictions and warnings.

## Methods

### Data

We used influenza A data for the Phoenix, AZ metropolitan area which has a population of ~4 million individuals. The period of the study was for September 1, 2015–June 27, 2016. The influenza data for 39 sites were provided by the Sofia® platform (Quidel, Inc.), a point-of-care immunoassay with an automated fluorescent reader for influenza A + B detection and for which HIPAA compliant data were wirelessly transmitted in real time. Sites with <100 positive influenza A tests and three consecutive days without a test during the epidemic were removed to eliminate sites that did not test consistently across time to improve the validity of the results. We excluded influenza B test results in this study due to the relatively low number of positives in Phoenix during the 2015–2016 influenza season. A weekly time-series of case counts by site was calculated from the daily data. The data for each site were also grouped into three broad age groups: <18, 19–45, and >46 years. The broad age divisions were necessary to retain a sufficient number of cases in each group for statistical analysis.

### Analysis

The goal of the study was to explore the synchronicity of influenza cases across the sites and age groups. Given differences in the number of tests performed at each site, we could not evaluate absolute differences in cases (i.e., positive influenza cases) over time to assess synchronicity. Instead, we used the Kolmogorov-Smirnov (KS) test to evaluate whether the temporal distribution of cases for each site and the temporal distribution of cases pooled across all sites were equal, adjusted for multiple comparisons using the Bonferroni correction (*n =* 9; α = 0.006). Similarly, we used the KS test with the Bonferroni adjustment to evaluate whether the temporal distributions of cases varied between age groups using the KS test (*n* = 3; α = 0.017); and whether distributions varied by age-group and site (*n* = 27; α = 0.002).

Although the series of KS tests performed determine the sites (and/or age groups) that are not synchronized, it does not provide information about when these differences occurred across time-series. Accordingly, to identify weeks within the outbreak during which cases at sites were not synchronized, we tested the null hypothesis that influenza cases were distributed proportionally across sites and time:

$$ \begin{array}{l}{\boldsymbol{\mathsf{H}}}_{\boldsymbol{\mathsf{O}}}:{\boldsymbol{\mathsf{P}}}_{\boldsymbol{\mathsf{i}},\boldsymbol{\mathsf{t}}}={\boldsymbol{\mathsf{P}}}_{\boldsymbol{\mathsf{i}}}^{*}\\ {}{\boldsymbol{\mathsf{H}}}_{\boldsymbol{\mathsf{A}}}:{\boldsymbol{\mathsf{P}}}_{\boldsymbol{\mathsf{i}},\boldsymbol{\mathsf{t}}}\boldsymbol{\ne}{\boldsymbol{\mathsf{P}}}_{\boldsymbol{\mathsf{i}}}^{*}\end{array} $$

where *P*_*i,t*_ is defined as the proportion of all cases at time *t* occurring at site *i*:

$$ {\boldsymbol{\mathsf{P}}}_{\boldsymbol{\mathsf{i}},\boldsymbol{\mathsf{t}}}=\frac{{\boldsymbol{\mathsf{C}}}_{\boldsymbol{\mathsf{i}},\boldsymbol{\mathsf{t}}}}{{\displaystyle \sum_{\boldsymbol{\mathsf{i}}=\mathbf{\mathsf{1}}}^{\mathbf{9}}{\boldsymbol{\mathsf{C}}}_{\boldsymbol{\mathsf{i}},\boldsymbol{\mathsf{t}}}}} $$

and *P*_*i*_ * is defined as the proportion of all cases occurring at site *i* during the study period :

$$ {\boldsymbol{\mathsf{P}}}_{\boldsymbol{\mathsf{i}}}^{*}=\frac{{\displaystyle \sum_{\boldsymbol{\mathsf{t}}=1}^{43}{\boldsymbol{\mathsf{C}}}_{\boldsymbol{\mathsf{i}},\boldsymbol{\mathsf{t}}}}}{{\displaystyle \sum_{\boldsymbol{\mathsf{t}}=1}^{43}{\displaystyle \sum_{\boldsymbol{\mathsf{i}}=1}^9{\boldsymbol{\mathsf{C}}}_{\boldsymbol{\mathsf{i}},\boldsymbol{\mathsf{t}}}}}} $$

In other words, if the cases are synchronized across study sites and 10% of cases across the study period occur at site *i*, then we would expect proportion of cases at time *t* for site *i* will be 10% of the total cases across all sites at time *t*. To do this, we used a bootstrapping approach. Specifically, for each time *t* we generated 1 × 10^6^ bootstrap samples of size *n*_*t*_ where *n*_*t*_ is the sum of cases across all sites at time *t*. We then calculated the proportion of cases in each bootstrap sample that occurred at each site (*i = 1, 2, … 9*) to create the bootstrap distribution for each site and time step (week). The non-parametric percentile-based approach was used to calculate the confidence intervals for the bootstrap distributions for each site and time, and to assess significance [15]. We used the same method to assess the significance of the cumulative proportion of cases by site. We used the Bonferroni correction to adjust for multiple tests (*n =* 43; α = 0.001). We performed the same bootstrapping approach on data aggregated by age, and by site and age. The results from the bootstrapping approach were compared with the results from a classical chi-square test and showed only minor differences. Accordingly, only the bootstrapping results are reported herein for simplicity.

Finally, we evaluated if the weekly cumulative proportion of cases across the nine sites were spatially autocorrelated using Moran’s I with the spdep package in R version 3.1.0 (R Core Team 2014). The k-nearest neighbors approach was used with the number of neighbors set to 3. We used the Bonferonni correction to adjust for multiple tests (*n* = 43; α = 0.001).

## Results

**Fig. 1:**
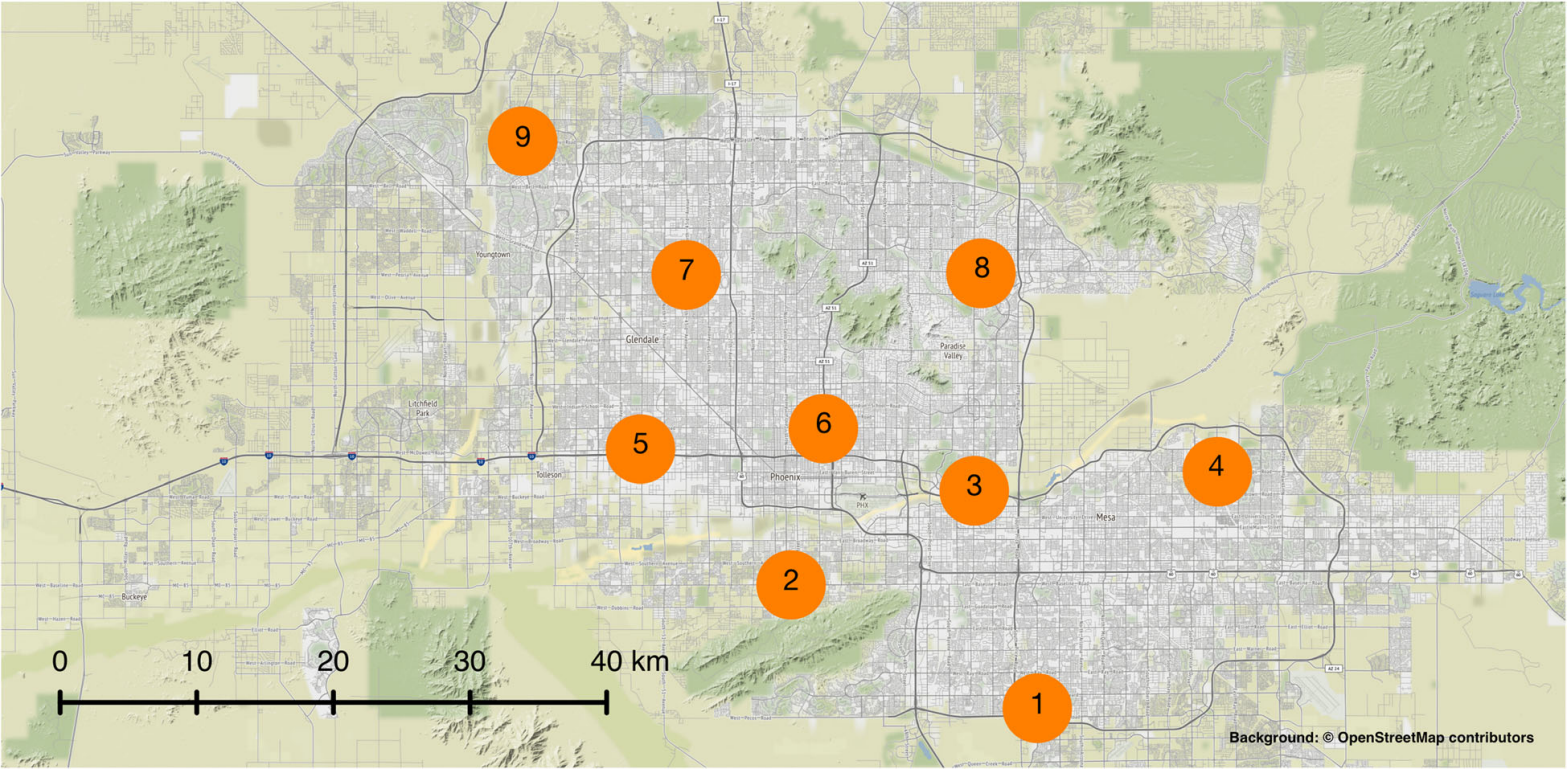
Influenza testing sites. The spatial distribution of the nine sites included in the analysis for the 2015–2016 influenza A epidemic in Phoenix, AZ

**Fig. 2:**
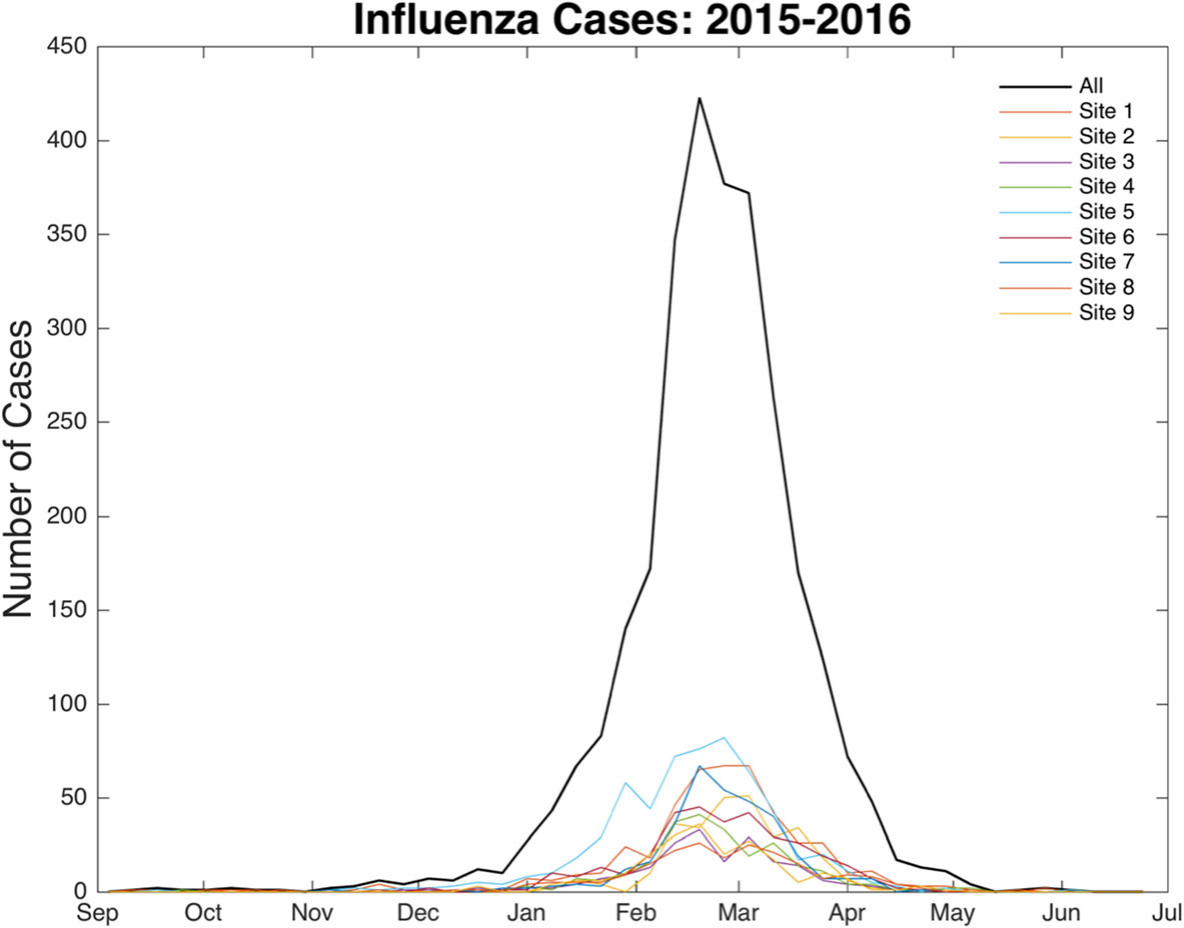
Influenza cases by week. The weekly number of cases across all nine sites. The *black line* represents cases aggregated across all sites. Cases increased rapidly in mid-December and peaked in mid-February to early-March. Differences in the timing of cases at some sites are evident in the plot

After removing sites with insufficient and inconsistent testing patterns, the dataset included 14,545 patient test results of which 3368 were positive for influenza A across nine sites in Phoenix, Arizona (Fig. 1). All nine sites were operated by the same health organization with the exception of Site 2. The first positive influenza A test occurred on September 10, 2015, but the rate of new cases across sites remained low until the second-half of December when cases increased (Fig. 2). Cases across the city peaked in mid-February to early March (Fig. 2). The epidemic was essentially complete by April 1, with 96% of all cases occurring before this time.

### Influenza activity by site and time

**Fig. 3:**
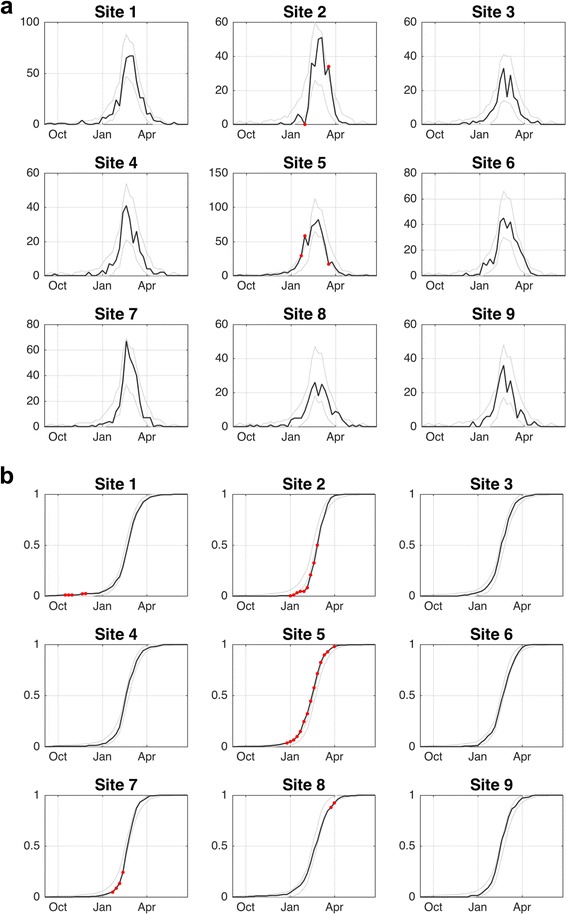
Influenza cases by site and week. **a** The weekly number of cases for each site. The *gray lines* indicate the 99.9% prediction interval for the null distribution. The *red circles* indicate weeks where the number of cases significantly (*p* < 0.001) diverged from the expected number. **b** Cumulative proportion of cases by site and week. The *gray lines* indicate the 99.9% prediction interval for the null distribution. The *red circles* highlight periods where the cumulative proportion of cases significantly (*p* < 0.001) diverged from the expected value

When we examine the timing of peaks in cases by site we observe several differences. Firstly, two distinct peaks are observed in the time series of cases at several sites, e.g., Sites 3, 6, 8 and 9 (Fig. 3a). Although the peak in mid-February dominated across most sites, the secondary peak in early March dominated at Sites 1 and 2 (Fig. 3a).

In addition to the difference in the peak timing of the epidemic, we also observed that the distribution of cases across time were not equal for all sites. For instance, the KS test indicated significant differences in the temporal distributions of cases for Sites 2 and 5 (*p* < 0.00001). Indeed, the proportion of cases occurring at Site 5 were greater than expected from December through early February, and this difference was statistically significant (*p* < 0.006) for several weeks in late January and early February (Fig. 3a). The cumulative proportion of cases was also significantly greater (*p* < 0.006) than expected for January-April for Site 5 (Fig. 3b). During this same period, the proportion of cases that occurred at Site 2 was lower than expected and this difference was statistically significant (*p* < 0.006) in early February, and the cumulative proportion of cases significantly (*p* < 0.006) lower than expected for January-March. To illustrate the magnitude of the differences in the timing of cases across Sites 2 and 5, by February 7 these sites had 8% and 31% of their total cases for the study period, respectively (Fig. 3b).

### Influenza activity by site, age and time

**Fig. 4:**
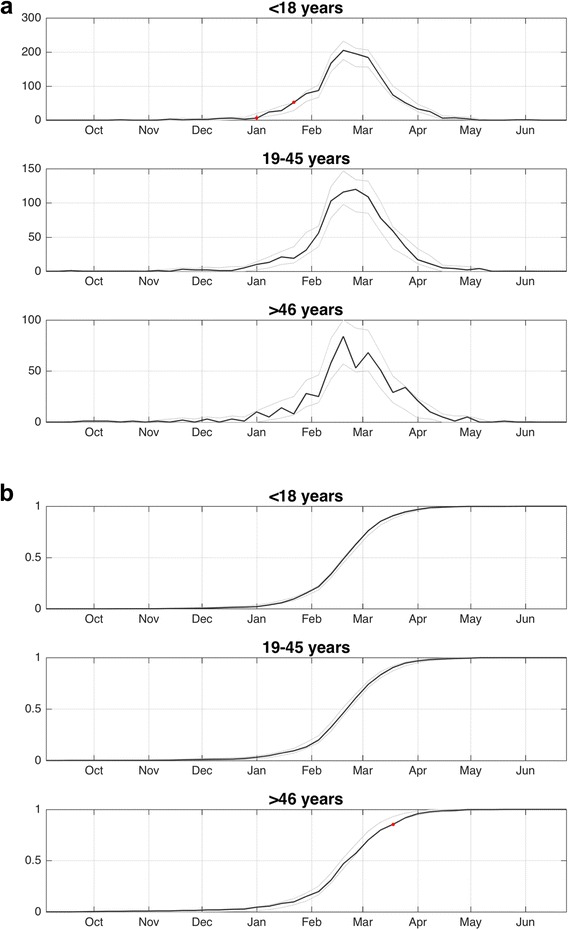
Influenza cases by age-group and week. **a** The weekly number of cases by age group. The *gray lines* indicate the 99.9% prediction interval for the null distribution. The *red circles* indicate weeks where the number of cases significantly (*p* < 0.001) diverged from the expected number. **b** Cumulative proportion of cases by age and week. The *gray lines* indicate the 99.9% prediction interval for the null distribution. The *red circles* highlight weeks where the cumulative proportion of cases significantly (*p* < 0.001) diverged from the expected value

**Fig. 5:**
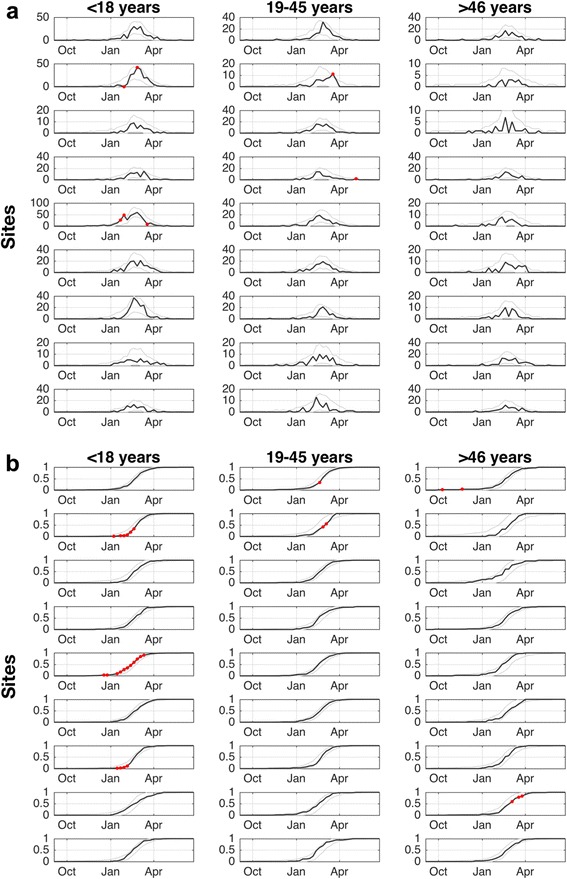
Influenza cases by age-group, site and and week. **a** Weekly cases by age groups, site and week. The *gray lines* indicate the 99.9% prediction interval for the null distribution. The *red circles* indicate periods where the number of cases significantly (*p* < 0.001) diverged from the expected number. **b** Cumulative proportion of cases by site, age and week. The *gray lines* indicate the 99.9% prediction interval for the null distribution. The *red lines* highlight periods where the cumulative proportion of cases significantly (*p* < 0.001) diverged from the expected value

Overall, there was little evidence that the timing of cases differed across age groups (Fig. 4). The KS test showed no statistically significant differences in the temporal distribution of cases across age groups. However, when we examined cases by age and site we observed that peak timing across age groups within some sites varied. For instance, in Site 2 the cases peaked in late-February among children, but not until mid-March for younger adults (Fig. 5a). We observe the surge of cases in late-January and early-February at Site 5 was primarily in children and younger adults, although a similar anomalous increase in cases in older adults occurred 2 weeks later. Finally, at the end of March and early-April we observed an increase in the proportion of cases occurring in older age group for Sites 4, 6, 8 and 9, but this increase was not significant after applying the Bonferroni correction.

We also found that the cumulative proportion of cases was significantly (*p* < 0.001) greater than expected for children at Site 5 for January-March, indicating that cases among children were weighted towards the beginning of the influenza outbreak at this site (Fig. 5b). However, at Sites 2 and 7 the cumulative proportion was significantly (*p* < 0.001) lower than expected in January, highlighting the different timing of cases across sites for the same age groups.

### Spatial autocorrelation

Moran’s I measures for the presence and intensity of spatial autocorrelation in spatial data, i.e., it tests the standard assumption that nearby locations are more similar than places that are further away. In this case, we measured the spatial autocorrelation in the cumulative proportion of cases for each week in the time-series. Moran’s I values varied across time, but the values were not significant after the Bonferroni correction was applied.

## Discussion

Our analysis indicates there was strong synchronicity of influenza A cases across sites and age groups within Phoenix, AZ during the 2015–2016 season. That said, we did observe several periods across sites where the number of influenza cases significantly diverged from the expected number. For example, one site experienced a significantly greater proportion of cases early during the ascending phase of the epidemic, whereas cases at another site lagged significantly behind other sites (Fig. 3). Further, the timing of cases within sites often varied by age group. For example, we observed an anomalous surge in cases among older adults across several sites during the descending phase of the epidemic (Fig. 4). We found no statistically significant evidence of spatial autocorrelation (i.e., spatial patterning of cases).

There are several factors that could engender differences in the timing of influenza cases across sites in a city. Although it is unlikely that environmental or air traffic patterns are relevant at this scale, workflow patterns within the city may create spatially structured contact networks that affect the progression of the epidemic across subpopulation within a city. Given evidence of heterogeneity of social contacts [16] and transmission rates across age groups [17], spatial variability of age structure within a city may also engender differences in transmission and case timing across sites. Another possibility is that immunity status varies significantly across subpopulations within a city. Indeed, Lessler et al. (2011) showed that detectable neutralization titers across five different locations in a single city in China (Guangzhou) were significantly different even after adjusting for age, employment status, vaccination history, household size and housing conditions [18]. Further, subpopulations with high levels of smoking, obesity, and poverty which are known risk factors for severe influenza infection [19–21], may also modify transmission efficiency across space. On the other hand, although we identified significant differences in the timing of influenza cases across sites in this study, the differences in the timing of cases were relatively minor. It is possible that these differences were the result of stochastic processes in the transmission system. If this is the case, this could suggest that the intensity of intra-urban population mixing is sufficient to largely synchronize infections despite variations in demographic and immunity patterns within a city.

The results of this study may be significant for researchers, clinicians and public health officials. This study suggests that the peak timing of influenza cases (and perhaps risk of infection) in a specific part of a city could occur several weeks after cases peak in other parts of the city. As such, clinics in the same city could experience maximum influenza case volumes at different times which could affect management of clinic staffs. Further, it provides a warning to researchers examining influenza dynamics. By aggregating data across populations such as a city, local variations in case rates could be masked which could detrimentally affect the results of studies, thereby inhibiting our understanding of influenza dynamics at local scales.

We were unable to identify significant spatial autocorrelation in the data, but this does not necessarily suggest that the variability in case timing is non-spatial. The absence could be due to an insufficient amount of data collected to detect a statistically significant signal. Further, it is possible that the spatial autocorrelation occurs at scales that are too granular for detection using the network of sites available for this study. Further, it should also be kept in mind that this study was limited to a single city and season due to data availability. It is possible that larger epidemics exhibit variability that is stronger and/or more spatially organized. Further, within a global context, Phoenix is a medium sized city with high levels of mobility among its citizens. The timing of influenza cases may be characterized by stronger variability at local scales in larger cities, particularly in less developed countries where population mobility can be limited [22].

We make several assumptions that could affect the results of this study. We assumed that utilization of the clinic sites is heavily weighted towards nearby residents. Given that this was a network of sites by the same provider (with the exception of a single site), it would seem that this is a relatively safe assumption although patients might also visit clinics near their workplaces. Also, we assumed that the health-seeking behavior of individuals with influenza and the consistency of testing by clinics was time invariant. Variability in either of these behaviors would affect the validity of the results and conclusions of this analysis. As such, although studies using secondary data may be able to establish strong evidence for variability in the timing and progression of epidemics across an urban area, large prospective cohort studies would be a preferred strategy for understanding these variations.

## Conclusions

To our knowledge, this is the first study to evaluate the synchronicity of confirmed influenza cases within an urban area. Our analysis shows influenza outbreaks are largely synchronized across sites and age-groups. That said, there were minor, yet statistically significant, differences in the timing of influenza cases at some points in the outbreak, especially when we examined cases by site and age. As local data sources become more numerous and long historical time-series are generated, the significance of intra-urban differences in the timing of influenza infections during influenza outbreaks will be better understood. Future studies could examine the relationship between the timing of cases and demographics, population mixing, social networks and transportation. Understanding the geographic scale at which influenza outbreaks are synchronized may be important for understanding transmission dynamics, to develop intervention strategies, and to generate prediction and warning systems that are spatially accurate.

## Abbreviations

KS: Kolmogorov-Smirnov
